# Sustainable Management of Medical Waste in Surgical Units: Operational Challenges and Policy Perspectives

**DOI:** 10.3390/healthcare14070954

**Published:** 2026-04-05

**Authors:** Ilie Cirstea, Ada Radu, Andrei-Flavius Radu, Delia Mirela Tit, Gabriela S. Bungau, Daniela Gitea, Bogdan Uivaraseanu

**Affiliations:** 1 Doctoral School of Biological and Biomedical Sciences, University of Oradea, 410087 Oradea, Romania; cirstea.ilie@student.uoradea.ro (I.C.); dtit@uoradea.ro (D.M.T.); gbungau@uoradea.ro (G.S.B.); 2 Department of Pharmacy, Faculty of Medicine and Pharmacy, University of Oradea, 410028 Oradea, Romania; dgitea@uoradea.ro; 3 Department of Psycho-Neurosciences and Recovery, Faculty of Medicine and Pharmacy, University of Oradea, 410073 Oradea, Romania; 4 Department of Surgical Disciplines, Faculty of Medicine and Pharmacy, University of Oradea, 410073 Oradea, Romania; buivaraseanu@uoradea.ro

**Keywords:** medical waste, Romania, sustainable development, surgical units, waste management, operating rooms, waste policies

## Abstract

Surgical wards constitute a significant contributor to global medical waste (MW), accounting for over one-third of total healthcare sector trash. Medical interventions produce hazardous, infectious, and potentially toxic byproducts, making effective MW management crucial, especially where current mechanisms are insufficient. Substantial disparities persist between high-income and low- and middle-income countries regarding MW infrastructure, enforcement, and adoption of safe, sustainable treatment technologies. Proper segregation, recycling, treatment, and disposal are key to protecting public health, environmental integrity, and promoting healthcare sustainability. Waste treatment technologies divide into thermal and physico-chemical processes, requiring thorough evaluation of advantages, disadvantages, and suitability for each waste type. This narrative review updates MW knowledge by synthesizing data from scientific literature, institutional documents, and regulatory sources. Key quantitative data indicate operating rooms generate up to 30% of total hospital waste, with recyclable materials representing over 40% of that volume. Improper segregation rates remain high, and incineration remains dominant despite sustainability concerns. The Romanian case study highlights progressive EU alignment, enforcing standardized MW classification, color-coded segregation, and specialized disposal protocols in surgical wards. Despite legal compliance, Romania is advancing incrementally, with systematic audits, digital tracking, and national outcome-based evaluations yet to be fully established. The Plastic Surgery Unit at Oradea County Emergency Clinical Hospital demonstrates good protocol adherence; however, strengthening data feedback mechanisms would enhance hospital-wide performance optimization and strategic waste reduction. Training and monitoring represent important areas for continued development. Coordinated professional engagement, modernized infrastructure, and enforceable audits are identified as critical priorities for improving MW handling in surgical environments. Future research should emphasize management innovation, evidence-based policy formulation, and a systematic strategy to achieve sustainable MW.

## 1. Introduction

In recent decades, waste production has reached record levels all over the world, becoming one of the most pressing issues affecting the contemporary global community. The consumer society, which formed in the last decades of the 17th century and developed throughout the 18th century, has now achieved its peak. This is due to several causes, including urbanization, population increase, flourishing economies, and the purchasing habits of consumers. It is predicted that by 2050, global production of municipal solid waste will have increased by around 70%, reaching 3.4 billion metric tons (t) [1]. Although medical waste (MW) is generally quantified separately from municipal solid waste streams and therefore cannot be directly expressed as a fixed global proportion, available evidence indicates that medical waste represents only a small fraction of total urban waste, commonly estimated at roughly 1–2% of the total municipal waste stream by mass. Despite this relatively limited share, MW requires specialized management because even a limited fraction may contain infectious agents, toxic chemicals, or radioactive materials, posing disproportionate risks to human health and the environment [2].

The majority of waste produced by healthcare activities consists of materials that do not present specific biological or chemical hazards and are similar in composition to conventional municipal refuse, representing approximately 85% of the total volume. In contrast, about 15% belongs to the hazardous fraction of healthcare waste, which may include infectious, chemical, toxic, or radioactive components and therefore must be managed through dedicated treatment and disposal procedures [3].

There is a growing demand for government agencies to offer proper waste treatment and disposal solutions due to the enormous amounts of generated waste. Although recycling rates have improved globally, they account for only about 19% of municipal solid waste, whereas approximately 30% is disposed of in controlled landfills and around 13% undergoes waste-to-energy treatment. In addition, nearly 38% of waste is still managed through uncontrolled disposal practices [4]. Open dump sites, particularly in underdeveloped countries, are another common method of waste disposal [5]. Wealthier nations tend to generate a greater volume of waste compared to their less affluent counterparts. However, they generally possess more effective waste management systems that aid in addressing these concerns [6].

The management of MW has a direct connection to several critical Sustainable Development Goals (SDGs), including SDG 3 (Good Health and Well-being), SDG 6 (Clean Water and Sanitation), and SDG 11 (Sustainable Cities and Communities). SDG 3 partly underscores the necessity of effective waste management to safeguard the public and healthcare workers from the risks of infectious diseases and hazardous chemicals, thereby ensuring safer environments. SDG 6 emphasizes the significance of preventing water pollution from MW, thereby protecting clean water sources through the implementation of effective waste treatment and disposal methods. SDG 11 is dedicated to the establishment of sustainable and resilient urban environments in which the burden on municipal systems is reduced and illicit dumping is mitigated through the implementation of efficient strategies, including efficient MW management. Healthcare facilities can make a substantial contribution to global sustainability initiatives by aligning their waste management practices with these SDGs [7,8].

The most environmentally detrimental industries and sectors globally [9] encompass energy (specifically electricity and heating) derived from fossil fuels [10], agriculture [11], textile production [12,13], food production and retail [14], transportation [15], manufacturing [16] and construction [17], technology [18], and the health field, which includes the pharmaceutical industry [19,20]. The sequence in which they occur is determined by the level of development in each country. The final negative effect is the same: waste, which contributes to the emission of greenhouse gases (GHGs) that drive climate change [21].

The substantial growth in medical technology and patient volumes over recent decades has been paralleled by a significant increase in the generation of MW [22]. Global MW generation rates vary considerably across regions, with median values ranging from 0.19 kg/bed/day in Africa and Oceania to 4.42 kg/bed/day in North America, and a global median of approximately 1.2 kg/bed/day for total MW and 0.46 kg/bed/day for hazardous waste [23]. Moreover, the findings show a gradual annual rise in MW production of about 2.11% [24].

In high-income healthcare systems, hazardous MW generation is estimated to reach approximately 0.5 kg per hospital bed per day, whereas facilities in low-income countries typically report lower levels, around 0.2 kg per bed per day [3].

ORs are recognized as major contributors to hospital waste generation, with estimates indicating that a single OR may produce up to approximately 2300 kg of waste each year. In addition, surgical suites can require up to six times more energy than many other hospital departments, highlighting their disproportionate environmental footprint within healthcare facilities [25]. Waste production in operating rooms (ORs) varies considerably across surgical disciplines and procedure types. Studies report that a typical orthopedic surgery may generate around 6.2 kg of waste per procedure, while total knee arthroplasty can reach levels of approximately 13.3 kg per operation [26].

Incorrect or illegal disposal of MW has the potential to pollute the environment and endanger human health. Methane and hydrogen sulfide are only two of the many toxic chemicals that can be released into the air when MW is stored in open-air environments [22]. The majority of those affected by MW are healthcare providers, including doctors and nurses, as well as cleaners, other hospital employees, visitors, municipal workers involved in waste transportation and disposal, and waste separators. Furthermore, MW has the potential to have detrimental effects on the surrounding ecosystem, air, soil, and water. Due to its particular features, MW requires specific handling throughout the waste management process, including isolated temporary storage, transportation, collection, and disposal. However, it is evident that these management practices are not implemented as a result of the increase in expenses [27].

Different countries, infrastructures, types of facilities, waste regulations, waste management strategies, technologies, and even income levels may all result in slightly different amounts and compositions of MW [28].

Infectious or hazardous waste coming from pharmaceutical waste and MW accounts for 27% of the total MW, while sharps and devices such as syringes and scalpels account for approximately 4% [29]. MW includes many items such as expired drugs, caps, chemicals, needles, disinfectants, bandages, and sharps [30].

The adverse effects of climate change on human health are well-documented, and it is noteworthy that the healthcare sector, despite its critical role in promoting well-being, significantly contributes to both waste generation and the production of GHGs. The manufacturing, shipping, and final disposal of obsolete medical equipment are major contributors to greenhouse gas emissions. Approximately 30% of all hospital garbage comes from ORs [31].

Two 5-day audits of the ORs at the Minneapolis Veterans Affairs Health Service, United States, revealed daily waste generation of 231.3 kg, broken down as follows: 2.79% biohazard waste, 3.88% blue wrap waste, 8.83% recyclable waste and 84.5% general waste. A systems approach to waste reduction in the OR and policy change to encourage environmentally conscious practices in the hospital setting could be achieved by research into the quantities and compositions of waste produced by various hospitals [32].

One-third of the healthcare system annual waste comes from ORs, making it the second greatest generator of trash in the United States [33]. With an increase in MW, it is becoming progressively more common to dispose of this type of garbage through combustion, composting, and landfilling, to mention a few of the most common means of disposal. Disposal methods for MW have been linked to numerous issues [34].

Epidemiological contexts like epidemics and pandemics exacerbate the challenges of managing the escalating volumes of hazardous MW in affected regions. The COVID-19 pandemic, in particular, highlighted significant issues in hazardous MW management. Prior to the pandemic, MW generation was relatively stable in healthcare systems. However, during 2020, a substantial rise was reported. Estimates indicate that daily biomedical waste increased from approximately 600 t before the pandemic to around 770 t during the peak period, with a considerable fraction associated with COVID-19-related healthcare activities. This increase, approaching 25%, was primarily driven by extensive use of personal protective equipment, diagnostic materials, and other infection-control consumables required for patient care, testing, and containment measures during the pandemic [35].

In Romania, the quantity of such waste increased from 8900 t in 2012 to 13,031 t in 2018, underscoring the growing complexity of this problem [36]. During the pandemic period, a further rise was observed, reaching roughly 21,903 t in 2021. Waste generation intensity also increased, from about 0.188 kg per hospital bed per day before the pandemic to approximately 0.330–0.440 kg per bed per day during 2020–2021. Similar trends were reported internationally as MW generation in Wuhan increased nearly sixfold during the outbreak, reaching about 240 t per day [37].

While these issues may pose serious threats to the natural world, they have an additional substantial impact on human society as a whole [38]. Reducing MW has many benefits, including saving money and helping the environment. According to the principles of the circular economy, it is more economical to use an item more than once before buying a replacement [39]. Saving energy and minimizing waste during manufacturing and shipping are two ways to help the planet. Furthermore, the healthcare industry is a paradigm for improving general public health [40].

The present narrative review was conducted in response to a notable scarcity of comprehensive analyses that specifically address MW management in surgical wards across diverse healthcare contexts, particularly in Eastern Europe. While previous literature has examined MW handling in general hospital settings or within broader healthcare systems, few studies have integrated technical, legislative, and practical perspectives in surgical environments, one of the most waste-intensive hospital sectors. This narrative review distinguishes itself by offering a multidisciplinary synthesis of waste classification, collection, and treatment methods, while also presenting a detailed case study from Romania, a country underrepresented in international waste management research. By examining Romania’s evolving regulatory frameworks alongside current practices at the surgical department level, this review offers granular insight into how European directives are operationalized in practice through a case from surgical departments, an angle often overlooked. Additionally, this approach highlights relevant data not only for decision-makers involved in shaping policies and legislation but also for healthcare professionals, enhancing their understanding of waste management practices.

## 2. Methodology of Research

The present narrative review, selected on purpose to integrate peer-reviewed evidence with context-specific insights from a Romanian case, synthesizes relevant data on MW management, with a particular focus on a context-specific surgical ward in Romania. This format was retained over a systematic review to allow the inclusion of heterogeneous sources (i.e., scientific literature, regulatory documents, institutional guidelines, and policy frameworks) essential for a comprehensive understanding of MW management in diverse healthcare and legislative contexts. Accordingly, no formal bias assessment or statistical synthesis was performed, as the aim was to provide a structured qualitative synthesis rather than quantitative approaches. By combining established scientific evidence with contextual evaluation, it offers an integrated perspective that goes beyond the limitations of secondary data alone. A comprehensive literature search was conducted across major scientific databases, including PubMed, Web of Science, ScienceDirect, SpringerLink, and Google Scholar. Additionally, institutional and regulatory sources such as Romanian Ministry of Health orders, European Union (EU) directives, national guidelines, conference proceedings, and official policy documents were consulted to ensure a multidisciplinary perspective.

The selection criteria targeted English-language papers and both primary and secondary sources (i.e., original research articles, review articles, book sections, official reports, directives, laws, technical documents, web page references from relevant institutions or organizations in the studied field) addressing MW generation, impact, management, and regulatory frameworks, as well as scientific literature related to MW legislation, sustainability, and the circular economy in healthcare. Studies were excluded if they were duplicates, unrelated to MW or clinical settings, or lacked relevant methodological or regulatory content.

Keywords and controlled vocabulary terms were applied where applicable, using combinations and Boolean operators: “medical waste”, “hazardous waste”, “hazardous waste AND hospitals”, “medical waste AND surgical wards”, “(medical waste OR healthcare waste) AND surgical ward”, “surgical waste management”, “medical waste AND Romania”, “medical waste AND Eastern Europe”, “medical waste management”, “waste segregation in hospitals”, “circular economy AND medical waste”, “sustainability AND medical waste”, “digitalization AND medical waste”, and “internet of things AND medical waste”.

After the identification stage, the retrieved sources were screened and organized thematically according to the main domains addressed in the review, including: (i) medical waste classification and regulatory frameworks, (ii) international experiences and comparative practices in surgical environments, (iii) operational challenges and waste reduction strategies in operating rooms, (iv) technological and digital innovations in waste management, and (v) policy and sustainability perspectives. This thematic organization enabled a structured qualitative synthesis of the literature and facilitated the integration of evidence from different healthcare systems and regulatory contexts.

Given the narrative nature of this review, the methodology does not follow the formal reporting standards of systematic reviews, and therefore does not include protocol registration, risk-of-bias assessment, or meta-analytical procedures. Consequently, the synthesis may be subject to selection bias and relies on the interpretative integration of heterogeneous sources. Nevertheless, the narrative approach was considered appropriate for the objectives of this study, as it allows for the integration of diverse evidence types, which are essential for understanding the complex and multidisciplinary nature of medical waste management in surgical settings, and allows for an update on the current state of knowledge in the field.

While no strict publication timeframe was imposed, preference was given to the most recent, high-quality, and policy-relevant sources, particularly those addressing current challenges, technologies, and regulatory developments. A total of 157 bibliographic references were selected based on relevance and their contribution to understanding MW management practices in surgical settings, with emphasis on legislative, technical, and institutional dimensions.

## 3. Types of Medical Waste

**Figure 1:**
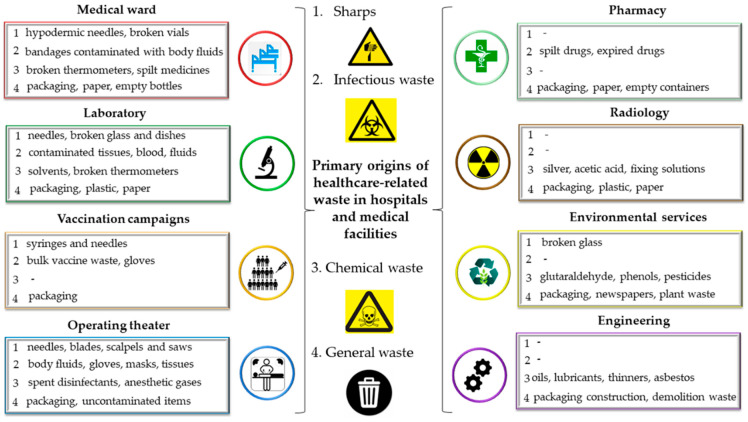
Departmental contribution to the hospital waste-risk profile (sharps, infectious, chemical, general). Hospital departments are mapped to the main waste classes, highlighting high-risk operational areas where improved segregation and staff training yield the greatest impact. Numbers (1–4) indicate the category of healthcare waste generated in each hospital unit: (1) sharps, (2) infectious waste, (3) chemical waste, and (4) general waste. The absence of certain numbers within some boxes indicates that the respective waste category is not typically generated in that specific department.

There are two main categories of MW: hazardous and general (non-hazardous). In contrast to hazardous MW, which can spread illness and harm the environment, non-hazardous waste poses no such threat [41]. Figure 1 illustrates the primary origins of MW in hospitals and healthcare facilities [42].

### 3.1. Classification of Medical Waste in Romania

For effective MW management in Romania, Annex no. 2 of Government Decision no. 856/2002, as amended, classifies waste into categories such as infectious, anatomical-pathological, chemical, pharmaceutical, cytotoxic, sharps, and non-hazardous. Hazardous waste is marked with an asterisk (*), and items not fitting defined categories are handled according to applicable laws [43,44]. Compared to international practice, Romania’s classification largely aligns with the European Waste Catalogue, though its practical implementation in surgical wards remains underreported in peer-reviewed literature [45].

Table 1 provides examples of the varieties of waste commonly encountered in medical activities, including the hazardous waste types described in Annex no. 2 to Government Decision no. 856/2002 and later additions [43].

### 3.2. Practical Romanian Experience Regarding Waste in the Surgical Wards in an International Context

The first regulation dedicated to the management of waste generated by medical activities was introduced in 2002 through Order of the Minister of Health no. 219/2002 [46], marking the beginning of Romania’s alignment with EU standards. Currently, multiple normative acts govern MW management, the most comprehensive being Order no. 1226/2012 [47,48], which covers all stages from classification to final disposal, in accordance with the European Waste Catalogue. These legal frameworks define specific responsibilities for healthcare institutions, economic operators, and public authorities. Healthcare units are required to implement protocols for the collection, segregation, storage, transportation, treatment, and final disposal of waste [47].

Country-specific studies illustrate the variability of healthcare waste generation rates across healthcare systems. For example, studies conducted in Brazilian hospitals reported general waste generation rates of 3.42 and 2.82 kg/bed/day, whereas a private hospital in Istanbul, Türkiye, reported 5.08 ± 12.26 kg/bed/day. At the regional level, median hospital waste generation reached 4.42 kg/bed/day in North America, 1.64 kg/bed/day in Latin America, 1.53 kg/bed/day in Asia, and 0.97 kg/bed/day in Europe, highlighting substantial international variability and supporting the use of standardized indicators such as kg/bed/day for comparative assessments [23].

In Romania, healthcare waste generated in surgical wards is segregated at source according to national regulations and the European Waste Catalogue, primarily into infectious, hazardous, and non-hazardous categories [32,33]. National monitoring data indicate a substantial increase in hazardous medical waste generation over the past decade. Quantities rose from approximately 8900 t in 2012 to 13,031 t in 2018 and reached about 21,903 t in 2021. Infectious waste accounted for the largest proportion of hazardous waste, representing between 84% and 96% of the hazardous fraction. Comparable indicators expressed per hospital bed highlight the same trend. Hazardous medical waste generation increased from about 0.188 kg/bed/day in 2018 to approximately 0.330 kg/bed/day in 2020 and around 0.440 kg/bed/day in 2021. These increases were largely associated with intensified infection-control practices [37].

**Figure 2:**
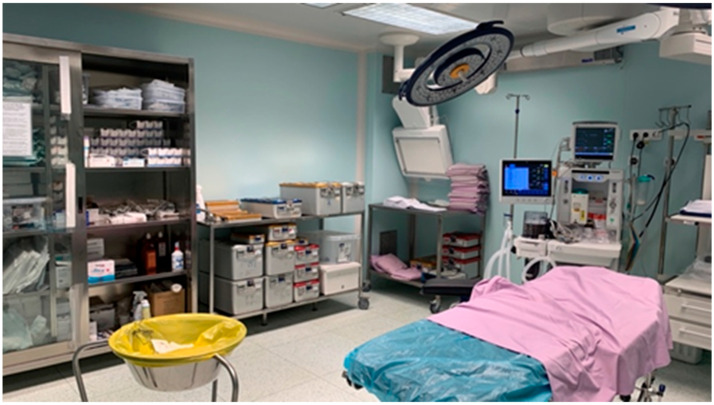
Operating room for plastic surgery: workflow layout showing consumables that generate medical waste and segregation at source via the yellow infectious-waste bag.

An example of regulatory-compliant approaches is presented for the Plastic Surgery Room (Figure 2) within the Operating Block of the Oradea County Emergency Clinical Hospital, located in Oradea, Romania. This facility exemplifies adherence to national standards in MW management and reflects the implementation of best practices in surgical environments.

The Bihor County Emergency Clinical Hospital in Oradea is the largest and most complex public healthcare institution in Bihor County, providing emergency, preventive, curative, and rehabilitation medical services. Currently, the hospital operates across seven locations with 63 departments and units, more than 48 medical specialties, 12 laboratories, and 1865 beds [49].

For contextual benchmarking purposes, reference values reported for Romanian hospitals can be used to approximate the potential scale of healthcare waste generation in such a large facility. Studies conducted in Romania suggest medical waste generation rates ranging between approximately 1.0 and 1.814 kg/bed/day [50]. Applying these reference values to the hospital’s reported capacity suggests a potential institutional waste generation range of roughly 1865–3383 kg/day, corresponding to approximately 680.7–1234.8 t per year. Considering the World Health Organization estimate that approximately 15% of healthcare waste is classified as hazardous [3], the potentially hazardous fraction would correspond to about 280–507 kg/day, or approximately 102.1–185.2 t/year. These figures should be interpreted as approximate contextual estimates rather than direct measurements, particularly as some of the underlying studies were conducted during periods of increased infection-control activity.

Beyond waste generation volumes, the capacity and geographic distribution of hazardous medical waste treatment infrastructure in Romania represent an additional structural factor influencing healthcare waste management performance, including in facilities located in the North-West region. National data indicate a total authorized treatment capacity of approximately 120,340 t per year as of 2018, of which about 79% corresponds to incineration and 21% to low-temperature thermal decontamination [37]. Under these conditions, regulatory compliance at the point of care, such as the color-coded segregation practices observed at the Oradea facility, represents a necessary but not sufficient condition for effective end-to-end waste management, since downstream treatment capacity, logistics, and regional infrastructure availability remain external constraints beyond the operational control of individual surgical units.

Together, these quantitative estimates and system-level capacity considerations illustrate how healthcare waste management performance depends not only on regulatory compliance at the point of generation, but also on the alignment between waste volumes, hazardous fractions, and the available treatment infrastructure, highlighting important implications for healthcare management planning, occupational safety, and environmental protection.

**Figure 3:**
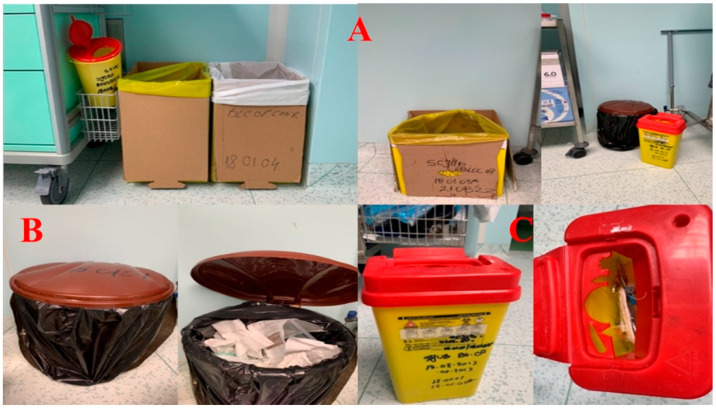
At-source segregation in a plastic surgery operating room (European Waste Catalogue-coded containers and color cues). Visual representation of labeled waste containers according to waste type and European coding standards. (**A**) Mixed container set with European Waste Catalogue labels applied at point of generation (e.g., 18 01 04 for non-infectious healthcare waste). (**B**) General recyclables stream (European Waste Catalogue 15 01 01 (paper/cardboard packaging)) collected in a lidded bin to prevent cross-contamination. (**C**) Yellow rigid sharps box for infectious sharps (European Waste Catalogue 18 01 01/18 01 03 *, The obligation to display waste collection instructions is marked with the * symbol) demonstrating compliant color coding and source-segregation of hazardous clinical waste.

**Figure 4:**
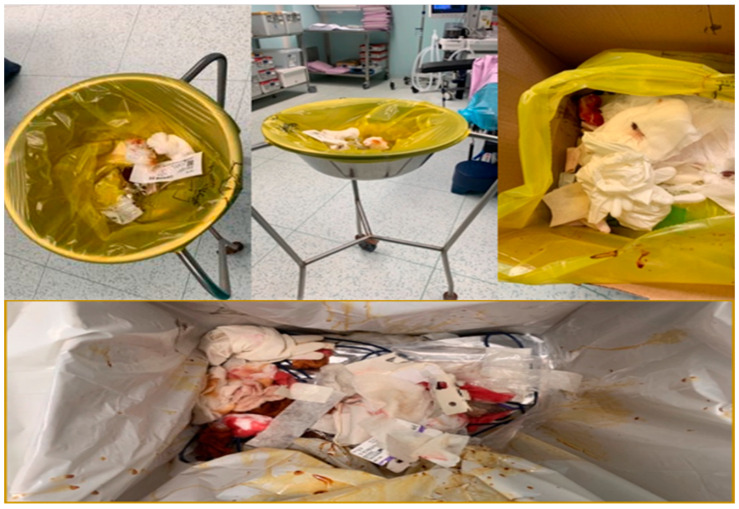
At-source segregation of infectious clinical waste (EWC 18 01 03 *, The obligation to display waste collection instructions is marked with the * symbol) in a plastic surgery OR. Composite images show the yellow-lined bins and infectious-waste boxes used for collection of infectious waste. The setup illustrates color-coded containment, leak-proof liners, and closed-lid handling to prevent cross-contamination during intra- and post-operative phases.

**Figure 5:**
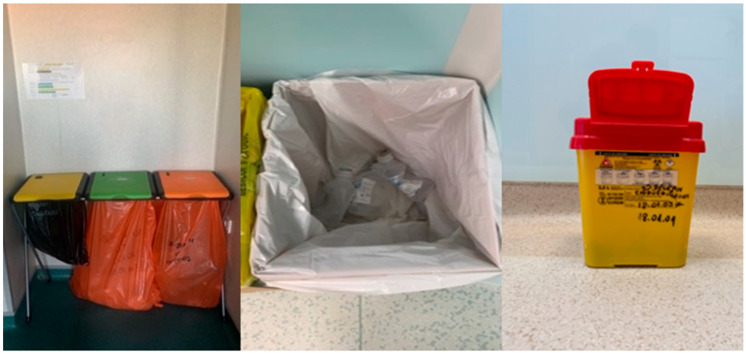
At-source segregation of non-infectious medical waste in perioperative areas. The figure shows non-infectious waste collection points (EWC 18 01 04): general bins lined in white or black, with clearly labeled containers for uncontaminated packaging and other clean disposables.

The obligation to display waste collection instructions is marked with the * symbol. The containers/bags intended for waste are shown in Figure 3, Figure 4 and Figure 5, inscribed with the corresponding codes.

In the process of waste collection, the correct codes are inscribed on containers and bags, and the colors that are utilized have significance as symbols (Table 2).

The containers intended for waste are inscribed with the corresponding codes. The code ^#^ is related to infectious waste, respectively waste that contains or has come into contact with blood or other biological fluids, as well as with viruses, bacteria, parasites and/or microorganism toxins, infusion sets with tubing, containers that have contained blood or other biological fluids, operating fields, gloves, probes and other disposable materials, compresses, dressings and other contaminated materials, dialysis membranes, plastic bags for urine collection, used laboratory materials, diapers that come from patients hospitalized in health facilities specific to infectious diseases or in infectious disease sections of health facilities, animal corpses resulting from research and experimentation activities, etc. The code * is related to hazardous waste, according to the provisions of annex no. 2 “List of waste, including hazardous waste” to Government Decision no. 856/2002. However, the collection area for materials used and contaminated during surgery, which do not constitute waste, is sent to the unit’s laundry and later to the sterilization room.

Data in the present case demonstrate compliance with EU directives through standardized color-coding and coded segregation. Nonetheless, the limited quantification of process indicators and the lack of systematic feedback mechanisms suggest that the system currently leans toward a descriptive rather than an effective waste reduction-driven approach.

While structural compliance with color-coded segregation protocols, as illustrated in Figure 3, Figure 4 and Figure 5, represents an indispensable regulatory foundation, infrastructure alone does not guarantee correct waste handling at the point of care. The behavioral and organizational dimensions of MW segregation are equally determinative of real-world outcomes. Evidence from behavioral research supports this perspective. A study applying the Theory of Planned Behavior found that attitudes, subjective norms, perceived behavioral control, and behavioral intention were significant predictors of healthcare waste segregation practices, with the model explaining 52.5% of behavioral variance and 66.7% when external variables were included. These findings indicate that segregation behavior is strongly influenced by psychological and contextual determinants beyond formal regulatory awareness [51].

Similarly, Tudor et al. identified staff habits, organizational constraints, and limited local control as persistent barriers to improved healthcare waste management practices within the National Health Service context [52].

Knowledge–attitude–practice assessments further confirm that procedural knowledge does not necessarily translate into consistent segregation behavior. In one hospital-based study, although approximately 80% of healthcare professionals demonstrated adequate knowledge of healthcare waste segregation procedures, only 56.4% exhibited good segregation practices, highlighting the influence of operational pressures and institutional conditions [53].

From an organizational perspective, compliance therefore depends not only on regulatory frameworks and infrastructure but also on training, supervision, workplace norms, and internal accountability mechanisms. In Romania, the current literature and institutional reporting largely emphasize infrastructure and legal compliance, while systematic assessment of staff attitudes, behavioral drivers, and ward-level organizational culture remains limited. Integrating attitudinal monitoring, targeted training programs, and safety-culture assessments alongside technical measures may therefore strengthen the effectiveness of existing segregation systems [54].

Comparatively, at Magee-Womens Hospital of University of Pittsburgh Medical Center in Pittsburgh, Pennsylvania, a targeted initiative launched in 2010 focusing on reducing regulated medical waste (RMW) in ORs and labor and delivery units led to a 47% decrease in RMW volumes. This translated into the diversion of approximately 28,795 pounds of waste and generated cost savings exceeding $89,000, highlighting the financial and environmental benefits of such strategic interventions. Furthermore, Inova Fairfax Hospital, a large medical center located in Northern Virginia with 833 beds, succeeded in reducing the volume of regulated medical waste produced in its ORs by 18.6% within a six-month timeframe [55].

In Romania, there is a predominant reliance on non-automated processing, insufficient digital traceability tools, and the continued dependence on incineration despite its environmental drawbacks [56,57].

Denmark has implemented structured reprocessing programs that demonstrate measurable environmental and economic benefits, including a documented 56% reduction in CO_2_ emissions for reprocessed ultrasound catheters and 13.5% cost savings in waste disposal through systematic reuse initiatives in healthcare facilities. Based on estimations performed by the working group using a model derived from an earlier study, Aarhus University Hospital has the potential to reduce annual expenditures on ultrasound catheters by approximately €330,000. Additionally, evidence from Sweden indicates that in-house reprocessing of specific medical devices could result in yearly cost savings of about €5.8 million [58]. Romania lacks such outcome-based assessments in surgical environments. This illustrates how structured reuse programs can simultaneously reduce emissions and costs, highlighting a model that could be progressively adapted in healthcare systems such as Romania where outcome-based evaluation of waste reduction strategies is still limited.

International experience also highlights the role of digital monitoring technologies in improving waste management efficiency. Sensor-based systems, smart bins, and Internet of Things (IoT)-enabled tracking tools allow real-time monitoring of waste levels, automated classification, and improved logistics planning. These technologies reduce transport requirements, improve traceability, and enhance regulatory compliance [59].

IoT-based waste monitoring systems enable real-time tracking of bin status, waste weight, storage conditions, and transportation routes, improving operational planning and regulatory oversight. These digital tools reduce manual handling, lowering occupational exposure to hazardous materials, while enabling data-driven management of waste flows. Additionally, continuous monitoring supports environmental control by tracking emissions, storage conditions, and treatment processes. However, current implementations remain fragmented, often monitoring only specific stages rather than the entire waste management chain [60].

Building on these capabilities, digital monitoring systems based on IoT architectures demonstrate measurable operational benefits in medical waste management. Sensor-integrated collection units capable of tracking waste level, weight, and storage duration enable real-time data transmission to centralized platforms, allowing automated alerts and workflow coordination among healthcare staff and waste management operators. Experimental validation of such systems showed high measurement accuracy, with weight detection errors below 1%, while automated notifications reduced the need for manual inspections and improved response time for waste removal. These capabilities support evidence-based management decisions, optimize collection schedules, and reduce unnecessary transport operations. In addition, minimizing direct handling of contaminated materials improves occupational safety, while more efficient waste monitoring contributes to safer disposal practices and reduced environmental contamination [59].

Beyond monitoring functions, IoT- and deep learning-based hospital waste systems can move the discussion beyond theory by linking digitalization to concrete operational outputs. IoT enables continuous monitoring of fill level, temperature, weight, location, and collection status, which supports real-time scheduling, route optimization, and more efficient allocation of waste-management resources. Deep learning contributes by improving visual recognition, segregation, and classification of waste items, thereby reducing sorting errors, cross-contamination, and inappropriate disposal. From a management perspective, these tools support data-driven decision-making and regulatory compliance; for occupational safety, they reduce direct handling of hazardous materials and limit exposure risks for workers; and for environmental sustainability, they can improve recycling, reduce waste volumes, and lower the ecological burden of disposal. Critically, cybersecurity, privacy, interoperability, training needs, and cost may limit implementation [61].

Furthermore, blockchain technology, integrated with IoT-enabled containers, is emerging as a transformative tool in biomedical waste management, enabling real-time monitoring, data exchange, and secure tracking from source to disposal. Using radio frequency identification tags, smart contracts, and consensus algorithms, blockchain ensures waste integrity, regulatory compliance, and transparency, while minimizing manual documentation errors [62,63].

Artificial intelligence (AI) enhances hospital waste management by enabling automated waste classification, predictive waste generation analysis, and optimized collection scheduling. AI models such as convolutional neural networks improve waste identification accuracy and reduce segregation errors, while reinforcement learning can optimize disinfection timing and storage management. These capabilities reduce manual handling and operational costs, support regulatory compliance, improve occupational safety by limiting exposure to hazardous waste, and contribute to environmentally sustainable disposal practices [64].

Building on these operational functions, AI enhances digital medical waste management by enabling predictive analytics and automated decision-support. AI models can forecast waste-bin filling levels, optimize collection schedules, and improve routing efficiency, reducing transportation costs and environmental impact. In practical implementations, explainable AI systems achieved high predictive performance (e.g., accuracy ≈0.92 with recall 1.0), enabling safer waste handling and timely collection. These capabilities support data-driven healthcare management, reduce occupational exposure risks, and improve environmental compliance in waste treatment operations [65].

At a strategic level, AI can strengthen digital medical waste management by providing decision-support tools that integrate operational, environmental, and regulatory data. AI-driven systems help identify priority waste streams, support circular-economy strategies, and guide management actions consistent with international guidelines. Empirical evaluations indicate strong alignment between AI-generated recommendations and healthcare professionals’ operational priorities, supporting informed decision-making. Such tools can improve waste governance, enhance environmental sustainability planning, and support safer, more structured management practices within hospital workflows [66].

Bluetooth positioning technologies track waste from source to disposal, enhancing transparency and preventing illegal diversion. Machine learning frameworks, such as predictive analytics models, improve volume estimation and route planning, achieving substantial reductions in waste generation, landfill use, and transportation needs while strengthening compliance and resource efficiency [62].

While some countries like Romania still rely on manual handling of MW, others have adopted advanced tracking systems. For example, South Korea uses radio frequency identification bags to trace MW from source to disposal, while Estonia monitors recycling behavior and has established two specialized centers for MW segregation and transport. Despite progress, digitalization remains more common in municipal than in MW management [60]. These findings suggest that technological adoption, rather than regulatory availability alone, remains the primary differentiating factor between advanced and transitional healthcare waste management systems.

Effective MW management increasingly relies on the digital transformation of both public and private waste management entities, an aspect that remains insufficiently integrated into current Romanian practices. A recent analysis of the German waste sector reveals that, although digitalization is gaining strategic importance, actual implementation often falls short of initial intentions. Many firms continue to rely on parallel analog and digital systems, leading to inefficiencies and operational burdens. For example, while 55% have adopted online sales channels, only 3% use them exclusively. Similarly, although container identification is digitized by 55% of firms, real-time tracking is implemented by only 7%. The primary focus remains on cost reduction rather than environmental or customer-centric innovations. Barriers include high implementation costs, lack of technical standards, and increasing concerns over data protection, especially post-General Data Protection Regulation. Despite these challenges, Germany demonstrates how long-established infrastructures can begin meaningful digital transformation. This shift is critical, as waste firms form the operational backbone of MW handling systems [67].

Although implementing digitalization in MW management can be complex and requires infrastructure, resources, and technological interoperability, this should not lead to the neglect of recycling. University Medical Center Utrecht generates approximately 2.5 million kg of waste annually, of which 40% is recycled. The hospital implemented source separation programs in ORs, labs, and administrative areas, supported by staff training and color-coded bins. In 2023, it introduced Sterilwave technology to decontaminate hazardous MW (e.g., needles, bandages) using microwave sterilization. This system converts waste into a powdery material, partly reused in the concrete industry, allowing the hospital to recycle up to 30% of contaminated waste, with a target of 70 t/year [68]. This is an example of good practice that Romania is aiming for.

Training plays a critical role in improving MW management and represents another essential pillar that requires stronger implementation at the national level in Romania. A quasi-experimental study showed that structured educational interventions significantly enhanced waste segregation practices in hospitals. Following training in a tertiary hospital in Spain, the monthly volume of MW decreased by 6.2%, with statistically significant reductions in infectious and pharmaceutical waste categories. This led to more accurate classification and cost savings of €125,205. The findings highlight that well-designed training programs not only reduce MW volume and associated risks but also offer measurable financial and operational benefits [69]. Similar findings were reported in India and Turkey, where structured education programs significantly reduced segregation errors and improved recycling rates [26,70]. Taken together, these studies indicate that behavioral interventions and continuous training represent some of the most transferable and cost-effective strategies for improving waste segregation performance across healthcare systems.

In Romania, despite the adoption of EU-aligned policies, systematic evaluation of their implementation in surgical environments remains limited, and there are no publicly available national audits or benchmarking mechanisms specifically targeting this subdomain. By contrast, the National Health Service in England, through its Health Technical Memorandum 07-01 (2023), requires all healthcare providers to report clinical waste volumes with at least 95% accuracy by 2024, to achieve defined segregation targets (i.e., 20% incinerated waste, 20% infectious waste, 60% offensive waste) by 2026, and to appoint dedicated waste management personnel with a workload equivalent to at least half of a full-time position in every institution. These requirements are accompanied by internal performance audits, routine compliance reviews, and integrated feedback loops that connect operational outcomes with governance structures. This framework offers a valuable model for Romania, where such structured evaluation and feedback mechanisms in surgical waste management are currently scarcely represented [71].

In light of the data relevant to the Romanian context, future directions in MW management should focus on improving digital infrastructure, developing advanced recycling strategies, and enhancing staff training programs in MW handling. These efforts must be closely integrated with structured feedback mechanisms to ensure continuous performance monitoring, adaptive learning, and long-term system efficiency.

## 4. Experience and Methods to Reduce the Impact of Surgical Waste

Although up to 85% of MW is non-hazardous and similar to household waste, it is often regulated as MW and subjected to special treatment, such as incineration [3]. RMW contaminated with blood, body fluids, or tissues poses risks due to its infectious, sharp, pharmaceutical, or radioactive nature and may require additional processing [72]. However, evidence assessing long-term environmental and economic outcomes of RMW management strategies remains limited.

The material composition of MW varies considerably by setting, country, and clinical context. Plastics constitute approximately 20–30% of total MW, with OR plastics including polyvinylchloride, polyethylene, and polypropylene as predominant polymer types. In Germany, only 1–3% of total hospital waste was categorized as infectious, with 75–95% resembling household waste containing neither body fluids nor sharps [73].

At the OR level, waste composition data from direct audits provide a more granular picture. Waste composition analysis in ORs indicates that textiles account for approximately 39% of total OR waste, plastics for 26%, paper and cardboard for 7%, with the remaining 27% comprising metals, glass, sharps, pharmaceutical residues, and anatomical or pathological waste. The predominant plastics identified are polypropylene, polyethylene, copolymers, polyurethane, and polyvinylchloride, of which the first three are theoretically recyclable. Notably, approximately 80% of OR waste is generated before the patient enters the room, rendering it non-infectious by default. Despite this, up to 90% of non-hazardous medical waste in the OR is incorrectly classified as hazardous, resulting in unnecessary high-temperature incineration costs [74].

These composition patterns are further corroborated by procedure-level waste audits. Procedure-specific audits of orthopedic and laparoscopic procedures recorded total quantified surgical waste at 84.4 kg, comprising 74.4% regulated MW, 19.4% non-regulated waste consisting predominantly of paper, plastic, cardboard, and wrapping materials, and 6.2% blue wrap, a 100% polypropylene sterilization material. When combined, non-regulated waste and blue wrap fractions represent approximately 25.6% of the total waste stream by weight and are theoretically recyclable. According to hospital data, only 24% of OR waste contains regulated medical waste, while non-regulated waste accounts for 59% and recyclable waste for 17%, highlighting the substantial recyclable fraction routinely misrouted to incineration streams [75].

Among individual material contributors, sterilization packaging represents a particularly significant waste stream. Blue sterile wrap, a polypropylene-based material used for instrument sterilization, constitutes approximately 19% of total OR waste and generates substantial disposal costs. Up to 80% of solid waste from a single surgical procedure is produced before the patient enters the room, originating predominantly from plastic packaging, with many products double-wrapped in large containers. Surgical suction canisters contribute up to 25% of biohazard waste from operating rooms, while reusable surgical linens, including gowns, drapes, and table covers, account for approximately 2% of total hospital waste. These observations align with international estimates indicating that the hazardous fraction of healthcare waste typically represents approximately 15% of the total waste stream [76]. However, comparable data from Eastern Europe and middle-income countries remain limited.

Global studies indicate that plastics represent approximately 20–25% of total MW, with considerable variation between countries, ranging from 12% in Peru to 47% in Italy. In Asia and the United States, annual medical waste generation is estimated at 5.5 and 5.3 million metric t respectively, with plastics contributing about 2.7 million metric t. Medical plastics, including bags, containers, and infusion sets, may release micro- and nanoplastics and leach chemical additives. These interactions remain insufficiently studied despite potential implications for therapeutic safety [77]. Micro- and nanoplastics may also cross biological barriers and induce oxidative stress, DNA damage, and inflammatory responses, highlighting the need for further real-world exposure studies [78].

Non-RMW does not require special handling [79]. Anesthesia-related waste represents approximately 25% of OR waste. In one study, weekly waste from unused endotracheal tubes and disposable laryngoscope components decreased by 62.6%, 54.7%, and 54.0% following an educational intervention, demonstrating the impact of staff training programs on waste reduction [80].

ORs generate 30% of hospital waste and consume a large share of hospital budgets (42% of income), with supply purchasing accounting for 56% of OR expenses. A pilot project recycling blue wrap saved $31,680 in avoided costs and could generate $5000 in revenue and $174,240 in annual savings if extended hospital-wide [81]. However, large-scale implementation remains limited outside high-income healthcare systems [82].

An evaluation of 38 studies found that 74% used pre-/post-intervention designs, but lacked formal implementation frameworks. Outcomes such as waste volume, cost, and energy use were assessed, while barriers included poor stakeholder buy-in, lack of resources, and data gaps. Only 23% of studies reported sustainable interventions such as audits, education, or policy changes, underscoring the need for structured waste reduction strategies and standard evaluation metrics [83]. This reflects a wider problem: inconsistency in outcome metrics (i.e., kg, cost, emissions), short follow-up periods, and geographic focus on high-income settings [84].

Waste segregation plays a critical role in optimizing healthcare waste management. This process categorizes waste according to contamination risk and recycling potential, allowing more efficient collection, treatment, and disposal. Segregation is performed manually at the point of generation within the healthcare facility, where staff separate RMW from non-RMW into designated color-coded containers, prior to collection and transport for treatment or disposal [85]. RMW requires special management and disposal because it contains contagious, pharmaceutical, sharp, or radioactive chemicals; it may even contain body fluids. Non-RMW, on the other hand, does not constitute a public health risk and can be disposed of using conventional methods [75].

Case analyses in surgical specialties demonstrate that large quantities of OR waste remain recyclable. For example, an otolaryngology study reported that approximately 80% of collected waste (out of 197.4 kg) was non-recyclable while 20% remained recyclable, supporting the potential environmental and economic benefits of institutional recycling initiatives [86]. This finding mirrors patterns in multiple high-income settings, but data from countries such as Romania are lacking.

Studies evaluating unused surgical supplies have also identified considerable financial losses associated with disposable materials. In neuro-interventional procedures, unused disposable items generated mean costs exceeding €500 per case, emphasizing the importance of improved supply planning and staff awareness to reduce unnecessary waste [87].

Institutional waste management programs have also demonstrated that structured policies, staff training, optimized transport routes, and dedicated supervision can significantly improve hospital waste handling practices [88]. However, the results are region-specific and lack broader generalizability or quantitative impact detail.

Surgical and maternity units remain major contributors to hospital waste generation, reinforcing the need for targeted waste-reduction strategies in these departments [89].

In a tertiary hospital anesthesia department with 35 ORs, implementation of improved waste segregation reduced RMW from 0.33 kg/case to 0.09 kg/case, resulting in annual savings of approximately $28,392. These findings demonstrate that behavioral interventions and improved segregation practices can significantly reduce both environmental impact and operational costs [90]. This suggests strong potential for cost reduction and environmental benefit through behavioral programs, but requires replication in diverse healthcare contexts.

Observational studies have shown that improper waste sorting remains common. In one evaluation, 57% of disposed waste was incorrectly classified, with 71% potentially recyclable materials incorrectly discarded. Lack of appropriate bins and misclassification by staff were identified as key causes of segregation errors [91]. This evidence highlights that infrastructure availability alone does not ensure correct waste segregation, emphasizing the need for integrated behavioral and organizational interventions within surgical teams.

Cost awareness among OR personnel may also influence waste generation. In surveys assessing cost awareness of surgical supplies, only 16.4% of respondents estimated item prices within 50% accuracy. Educational initiatives improving cost awareness among OR staff may therefore contribute to reducing unnecessary material consumption and associated waste [92]. This finding suggests that financial awareness among clinical staff may represent an underutilized lever for reducing both operational costs and waste generation in surgical environments.

Educational gaps remain evident among healthcare professionals. A cross-sectional assessment in surgical departments found that adequate waste management knowledge was present in only 36.8% of physicians, 27.4% of nurses, and 32.1% of housekeeping staff, highlighting the need for systematic training programs [93].

**Figure 6:**
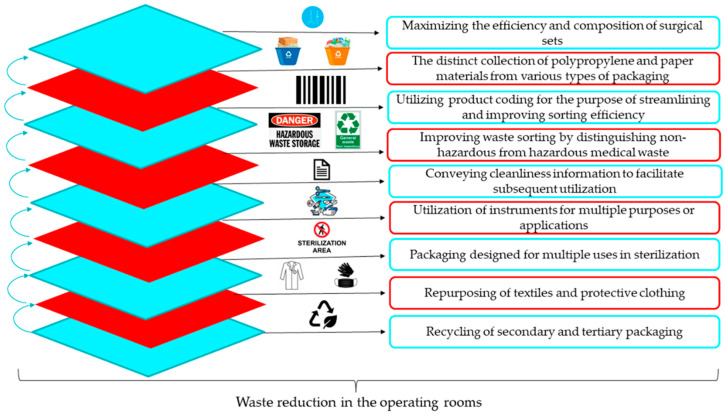
Intervention levers for operating room waste minimization: set optimization, hazard segregation, coding, cleanliness signaling, multi-use/repurposing, and recycling.

Design-based strategies also contribute to waste reduction. Reuse and circular economy principles such as reduce, reuse, recycle, and repurpose represent key approaches for improving OR sustainability. However, implementation of these strategies remains uneven, particularly in lower-income healthcare systems [94]. Figure 6 shows several intervention strategies that can be used in order to reduce waste in the OR [74].

Finally, surveys among surgeons indicate strong awareness of surgical waste issues. Approximately 90% of respondents recognized sterile surgical waste as a problem, and 95% expressed willingness to modify clinical workflows to reduce waste. However, barriers such as lack of time, limited awareness, and insufficient institutional support remain significant challenges [31].

## 5. Medical Waste Management

Adequate MW management ensures that all MW is properly handled from the point of generation through its collection, transportation, storage, treatment, and ultimate disposal [95].

The increase in MW is driven by environmental and socioeconomic factors, such as CO_2_ emissions, mortality rates, life expectancy, and rapid urbanization [96]. MW generation data remains scarce across countries, yet available estimates reveal notable variations. In China, annual per capita MW production ranges between 0.7 and 1.5 kg, averaging 1.0 kg, while Greece and Lebanon report 0.70 kg and 1.42 kg respectively. South Korea records significantly higher levels at 7.2 kg per capita annually. In 2023, the National Health Service providers in England generated approximately 156,000 t of MW, whereas Türkiye’s output was forecasted at 143,500 t, with projections reaching over 211,000 t by 2030. These trends reflect the impact of expanding healthcare systems and industrialization on MW volumes [97].

Waste separation is broadly acknowledged as the essential first stage of waste management. MW must be properly separated from other trash at all healthcare institutions. Various members of the medical staff, such as nurses, doctors, scientists, and technicians, can execute segregation immediately, at the point of waste formation, such as in ORs, laboratories, wards, or treatment facilities [98].

Beyond waste treatment technologies, the operational chain of MW management also involves several critical stages, including temporary storage, collection, and transport within and outside healthcare facilities.

Effective MW management requires systematic approaches to temporary storage, collection, and transport, each governed by distinct regulatory requirements and operational constraints. Optimal waste storage within healthcare facilities requires physical separation of waste categories into dedicated areas, hazardous waste in one room and special or household-equivalent residues in another. Storage areas must comply with regulatory space requirements, maintain daily general hygiene and weekly complete disinfection, and be accessible only to authorized personnel. Sharp waste must be packaged in rigid containers to minimize human contact, with box arrangement optimized to maximize space utilization and reduce accumulation-related exposure risk. Investment in refrigeration equipment can extend permissible storage duration by maintaining temperature-controlled conditions, reducing collection frequency requirements for facilities with limited logistical capacity. Waste flows through two internal zones, the generation zone, where source segregation occurs into color-coded containers, and the storage zone, where packaged waste awaits authorized external collection [99].

Permitted storage durations vary considerably by context and facility type. Temporary storage duration is directly linked to facility size: in hospitals with approximately 250 beds, waste must not be stored for more than 8–10 h, while nursing homes are permitted up to 24 h [100].

In Europe, temporary storage ranges from 28 to 90 days, while in the United States large-quantity generators may store waste up to 90 days and small-quantity generators up to 180 days. In Eastern Europe and Africa, temporary storage may be indefinite due to infrastructure limitations [101]. Storage bags and containers must be securely closed to prevent spillage, and the storage room must be physically separated from patient care areas [100].

Beyond temporary storage conditions, effective waste management also depends on structured and safe collection procedures. MW collection should occur at minimum once daily to prevent infectious accumulation, with personnel equipped with appropriate protective gear throughout the process [73]. Color-coded containers are mandatory at each point of waste generation, labeled by ward or department, and loading must not be performed manually to prevent handler exposure [100].

Category-specific collection protocols differ substantially between healthcare systems. In Germany, sharps must be placed immediately in leak-proof closable containers at the point of generation, anatomical waste must be refrigerated prior to transport, and infectious waste must be collected in biohazard-marked containers for transfer to authorized incineration facilities by licensed operators. In contrast, in Egypt, collection relies on non-specialized trolleys with no protective measures, with waste stored in utility rooms or intensive care units for an average of four to eight days before disposal [73].

Following collection, the safe transport of medical waste represents a critical stage linking healthcare facilities with treatment and disposal infrastructure. MW transport involves two interdependent risk dimensions: occupational risk during temporary storage at healthcare centers and public risk during road transport to treatment facilities. Dedicated collection vehicles operating on periodic weekly schedules service healthcare centers based on storage capacity constraints and waste accumulation rates. Infectious waste requires daily treatment at ambient temperature or weekly treatment if stored below 5 °C. Large hospitals may require daily collection, while smaller facilities may be served once or twice weekly. Minimizing combined transport and occupational risk simultaneously yields a 26.25% reduction in total risk compared to cost-minimization routing strategies, demonstrating that risk-integrated scheduling substantially improves operational safety outcomes [102].

Transport vehicles or trolleys must be fully covered, and designated routes with minimal public traffic must be used [100]. Each hospital must be served by a dedicated vehicle, with vehicles returning to designated disposal sites after collection. Outsourcing collection and transport to authorized contractors represents a cost-effective strategy, particularly in densely populated urban areas where municipal capacity is insufficient [103].

During exceptional circumstances such as pandemics, transport logistics become even more complex and require coordinated system-level responses. Infectious medical waste transport during pandemics involves a three-tier reverse logistics chain linking hospitals, temporary transfer points, and disposal centers. Cross-regional transportation strategies from hospitals to transfer points are recommended to optimize costs, infection risk reduction, and job creation simultaneously. Differentiated transportation schemes, routing strongly infectious and weakly infectious waste to separate transfer points, improve resource allocation and reduce cross-contamination risk. Source separation at the point of generation is essential for operational efficiency throughout the transport chain [104].

A multilevel intervention implemented in a Spanish surgical department demonstrated that improving the segregation of non-hazardous MW significantly reduced the institution’s carbon footprint, by 85% weekly. This approach serves as a scalable model for other healthcare settings seeking to reduce environmental impact through optimized waste segregation [105].

A smart medical waste management system integrates IoT-enabled trolleys with sensors for waste level, weight, and storage time, enabling authenticated lid control and real-time tracking. Data is transmitted to a cloud platform and web application, which notify stakeholders for timely action, ensuring transparency, safety, and compliance. This approach significantly improves operational efficiency, worker safety, and environmental protection in medical waste handling [59].

SmartMedWaste is an integrated IoT- and AI-enabled system designed to optimize medical waste management through automated segregation, monitoring, and compliance. This end-to-end framework reduces human error, improves resource allocation, and supports data-driven decision-making, representing a significant advancement in safe, efficient, and environmentally responsible medical waste handling [106].

Following the generation and segregation of MW, storage and collection represent another critical point of exposure, particularly for MW handlers. In LMICs, improper sharps disposal, insufficient use of personal protective equipment, and lack of formal training significantly elevate the risk of infection. Studies from Ethiopia, Sudan, and Brazil report alarmingly high rates of needlestick injuries and exposure to blood-borne pathogens such as hepatitis B virus and HIV. In contrast, high-income countries show lower infection risks due to more stringent safety standards, broader vaccination coverage, and structured handling protocols. Enhanced protective measures and education are urgently needed [107].

Timely transfer and transportation of medical waste, ideally within 48 h, is essential to prevent cross-infection and environmental contamination, especially in dense urban areas [108]. During pandemics, infectious medical waste management requires efficient transfer and transport strategies. A bi-level optimization model applied to a real case in Wuhan showed that combining augmented ε-constraint and Pareto optimization effectively reduces infection risk and cost. The results highlight the importance of cross-regional transportation and adaptive disposal center placement to support sustainable, responsive reverse logistics systems under uncertainty [104].

A study that explored MW management indicated that the environmental and human health consequences could be mitigated by the deployment of an effective MW management system. The following steps, designed to cut down on waste, were proposed: use of contemporary medical techniques and equipment can help cut down on waste at the source; sorting waste at the source into different bins based on their category; final disposal under proper conditions, by only licensed operators, to lessen the ecological footprint of the process; staff participating in waste management should be given opportunities for professional development; hazards should be identified, and the required precautions should be taken; educating people on the risks associated with MW that has not been properly managed [109].

In five Tehran hospitals, medical waste generation averaged 3126 kg/day, with 52.53% infectious and 44.67% common waste. Management costs were dominated by disinfection (67.3%), followed by transport (16.5%) and collection (12.3%). Scenario analysis showed that increasing infectious waste segregation and using on-site disinfection substantially lowered costs, by up to 50% and 25%, respectively, while reducing pollutant emissions, confirming this combined approach as the most cost-effective and environmentally sound strategy [110].

Waste treatment methods are selected based on health risks, environmental impact, economic cost, and social acceptance. Common techniques include incineration, autoclaving, microwave irradiation, and chemical disinfection. In lower-income countries, limited resources often result in reliance on high-emission, low-tech solutions [111]. Strategies for reducing MW include minimizing source generation, guiding hospital supply use, segregating waste into categories (infectious, chemical, radioactive, sharps, etc.) before disposal, and promoting recycling or reuse [112]. The disposal of the 20–33% of MW that comes from the ORs is particularly expensive [113]. Effective MW management hinges on proper segregation at the point of generation [114,115]. However, the effectiveness of these strategies depends largely on consistent implementation and monitoring mechanisms, which remain unevenly developed across healthcare systems.

The waste removal procedure consists of three phases: pretreatment, treatment and disposal. Grinding, liquid-solid separation, shredding, agitation, mixing, and crushing are some of the mechanical processes that are primarily included in pretreatments. However, these steps do not eradicate infectious microorganisms or disinfect equipment, despite the fact that they have the advantage of reducing the overall volume of trash [116].

Different types of waste require different methods of treatment, which can include steam sterilization, microwaving, reverse polymerization, dry heat treatment, dry and wet carbonization, converter technology, bio converter, incineration, pyrolysis, gasification, irradiation, chemical disinfection, immobilization [117]. However, comparative assessments of these technologies across contexts are limited and often Western Europe. To effectively identify and manage waste, segregation plays a crucial role. The best way to distinguish between different types of trash is to sort it according to the color of the container [118].

Incineration, or the annihilation of waste by burning, eliminates dangers by reducing waste’s volume and mass and turning it into ashes. If an incinerator is not built, maintained, and run correctly, it will release harmful chemicals into the air. Dioxins and furans, both of which are carcinogenic, are released into the air when incinerators run at low temperatures [119].

Medical equipment is sterilized in autoclaves by subjecting it to high temperatures and pressures for extended periods of time. Sterilizing medical equipment in an autoclave has been standard practice for nearly a century. Before MW is delivered to a landfill, it is treated in an autoclave to kill any bacteria that may be present. Autoclaves may be easily scaled to fit the demands of any medical institution and may be utilized to treat as much as 95% of MW [120].

By heating waste to extremely high temperatures, harmful microorganisms are killed [121]. This strategy is typically employed for substantial quantities. Heat exchangers or a steam jacket may be used to warm the vessel in which the collected liquid waste is stored. The waste’s temperature and treatment time are both determined by the present germs [120].

The following step is a chemical process treatment. Disinfectants, ozone therapy, and alkaline hydrolysis are the mainstays of this method. Hospital culinary waste, along with various digestible organic and placental waste, can be broken down through vermicomposting and composting. Burial of pathological waste allows for its natural decay, which is an example of a biological process. Municipalities should collect non-hazardous waste on a regular basis for recycling, and the facility shall transport this material to public landfills [85].

Microwave disinfection involves the use of high-power microwaves at low temperatures to cause the depolymerization of organic compounds and the breakdown of bacteria. High-priced microwaves can be used in tandem with the more cost-effective cremation and autoclave methods of disinfection [122].

Open burning and incineration are the most hazardous disposal methods, especially in developing countries, due to emissions and human health risks. Although landfilling avoids air pollution, it occupies land and can release hazardous chemicals into soil and water. However, if managed properly, landfilling remains a cost-effective option with lower greenhouse gas emissions compared to poorly operated incineration [116].

Conversely, steam sterilization and microwaving are financially feasible and have a diminished environmental impact in comparison to chemical processing, incineration, and landfilling [123]. Moreover, converter technology is a novel process that consolidates a variety of operations, including cooling, garbage compaction, pasteurization, grinding and pulverization, dehydration, and sterilization, within a single unit [124].

The bio converter system sanitizes MW by utilizing a solution of enzymes. The final product is a sediment consisting of solid waste that is transported to the sanitary landfill, and the wastewater is extracted for sewage discharge [125].

The process of waste immobilization is the act of embedding or encapsulating refuse to convert it into a compact form [126]. Bituminization, cementation, and vitrification are the primary immobilization technologies that are implemented. Vitrification is the most frequently employed technique during the cleanup of radioactive waste at high levels [127].

However, the selection of technologies is influenced by a variety of factors, including the waste type, technical reliability, health and security considerations, reduction in waste mass and volume, compliance with regulations, environmental emissions and residues, the size of the system and the necessary space, the level of automation, the loading capacity, and cost [117].

Poorly managed landfilling or burial of MW poses serious environmental and public health risks, such as leaching of toxic substances into soil and water. Ensuring safety requires hydro-geological assessment, access restrictions, regular covering, and prevention of scavenging or run-off [128].

A recent life cycle assessment (LCA) of hospital waste management in Isfahan, Iran, provides quantitative evidence on the environmental performance of alternative treatment strategies for infectious and non-infectious waste streams. Isfahan hospitals generate ~20 t/day of waste. TRACI 2014 LCA shows composting and material recovery lower smog precursors by 126.79 and 129.02 kg O_3_-eq/t and cut fossil fuel depletion by 255.04 and 471.97 MJ-surplus/t versus incineration. For infectious waste, chemical disinfection has lower global-warming potential (859.89 kg CO_2_-eq/t) than pyrolysis (1483.15). A scenario prioritizing composting plus recovery minimizes ecotoxicity (Cd 0.33 g/t, Ni 16.23 g/t to air; As 0.003 g/t, Cr 0.28 g/t to water), supporting circular-economy management with regulatory and training investments [129]. These findings reinforce the importance of integrating environmental impact assessment tools into healthcare waste policies in order to support evidence-based decision-making.

To accelerate circular economy transition in the MW sector, strategies should target both quality and quantity of recycling. These include: (1) adopting waste category-level performance monitoring using multi-indicator frameworks (i.e., recycling rate, recycling circularity, dynamic circularity performance index); (2) improving waste classification accuracy to avoid over-incineration of non-hazardous waste; (3) expanding recycling capacity and upgrading to higher circularity level processes; (4) implementing policy incentives for high-quality recycling; (5) reducing the single-use mindset through material redesign and safe reuse protocols; and (6) integrating data correction methods for long-term trend evaluation, ensuring performance metrics remain valid during waste surges, such as pandemic-related increases [130,131].

The disposal of medical waste (MW) from major infectious diseases presents distinct challenges compared to general MW management, due to its higher infectivity and rapid generation during outbreaks. Safe disposal requires stringent protocols, rapid scalability, and integrated, intelligent management systems. Sudden surges in MW volumes, as seen during the COVID-19 pandemic, often exceed existing treatment capacities, particularly in early phases. Disposal methods must ensure complete inactivation of pathogens under strict environmental and temporal constraints. Moreover, limited infrastructure, especially in low-resource settings, exacerbates risks of improper handling and secondary transmission. Therefore, comprehensive, real-time evaluation systems are essential to identify safety risks, support closed-loop management, and guide timely interventions throughout the MW disposal process during epidemics, ensuring both public health protection and environmental safety [132].

The MW resulting from the preceding processes is subsequently transported to an appropriately designated landfill site for final disposal in accordance with regulatory standards. Leachate from waste decomposition pollutes both groundwater and surface water, while trash incineration releases gases into the atmosphere. As a result, it is crucial to find ways to reduce the amount of garbage directed to the landfill [122]. Moreover, mixed MW, including plastic, glass, and can be pyrolyzed at 500 °C to generate liquid fuel (i.e., pyrolysis oil), as demonstrated by Fang et al. [133]. Moreover, medical syringe waste can be converted via pyrolysis into oil with a 71% yield, offering a potential alternative fuel for compression ignition engines [134].

Research on pharmaceuticals often focuses on the first three phases of the product’s life cycle (development, production, and patient usage). After the expiration of the shelf life of various pharmaceutical and sanitary items, an investigation intended to conduct research into the final phase of the product’s life cycle, recycling. The subjects in the study proved to be quite receptive, with a rise from 1.1% to 87.3% in the proportion of pharmaceutical wastes returned at pharmacies throughout the course of the study’s first six months. Customers and patients are more likely to dispose of unwanted items in a targeted manner when pharmacies provide a designated trash can for old or unused medication. Because of the massive amount of waste people produce, steps to reduce drug use and, by extension, waste output are needed [135].

Another research evaluated how well residents of Bihor County, in western Romania, comply with legal requirements for the final elimination of pharmaceutical wastes produced by in-home treatment. In order to assess health, medication use and storage practices, and procedures for disposing of unused or expired drugs, a total of 739 participants completed a questionnaire. Based on the findings, it is clear that public information initiatives are greatly needed. Such campaigns could be carried out, for instance, by the specialized staff from pharmacies and by using instructive advertisements [136].

In Romania, pharmaceutical and MW disposal in pharmacies faces systemic issues such as high handling costs, unclear legislation, and limited staff awareness. Pharmacists and technicians would benefit from legal training and awareness campaigns, while a clearer legal framework would help assign responsibilities and reduce the burden on pharmacies, improving public health and environmental outcomes [56].

To facilitate cross-study comparison and address the heterogeneity of reported metrics, Table 3 summarizes representative studies on medical waste generation, management interventions, and the main lessons learned.

**Figure 7:**
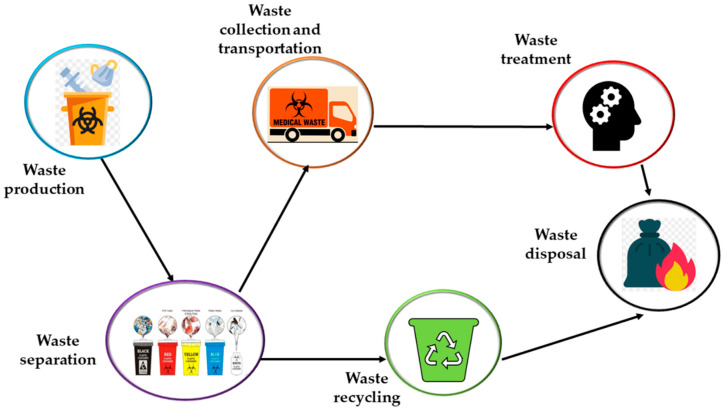
Process map of clinical waste management highlighting segregation-driven pathways and circular recovery options.

Figure 7 illustrates the complete waste management process, from generation to disposal [73].

### Comparative Data of International Practices

International MW management practices reveal significant variations in regulatory frameworks, treatment technologies, and operational efficiency across healthcare systems globally. WHO’s comprehensive guidance document “Safe management of wastes from health care activities” (2014) addresses regulatory frameworks, planning issues, waste minimization and recycling, handling, storage and transportation, treatment and disposal, yet implementation varies considerably among countries [137].

Significant disparities exist in MW management between high-income countries and those in transition. European nations like Sweden and Germany have developed robust systems prioritizing recycling, emission control, and safe incineration. In contrast, while China has introduced regulations for hazardous waste treatment through sterilization, incineration, and landfilling, enforcement remains less rigorous. Meanwhile, countries such as India and various African states struggle with limited infrastructure, leading to improper disposal practices and environmental hazards. These contrasts underscore the need for context-specific strategies, enhanced regulatory enforcement, and investment in sustainable MW infrastructure in low- and middle-income countries to bridge the implementation gap [138]. From a comparative perspective, these differences demonstrate that successful MW management systems combine regulatory frameworks with operational monitoring and technological support.

The EU employs the European Waste Catalogue classification system under Directive 2008/98/EC [139], which differs from approaches in other regions. The United States Environmental Protection Agency provides Model Guidelines for State Medical Waste Management [140], allowing individual states significant flexibility in implementation. According to World Health Organization data from 2021, only 61% of hospitals globally had basic health-care waste services, with the situation far worse in fragile contexts where only 25% of health facilities had basic MW management services in 2023 [3].

Treatment technology selection varies significantly based on economic development and infrastructure capacity. Incineration remains the dominant method globally despite sustainability concerns, as noted in the original research findings. According to European Environment Agency reports, improper collection practices can lead to a 30% increase in environmental and health risks [141]. Advanced autoclaving and microwave systems are predominantly adopted in developed healthcare systems, while developing countries often rely on less sophisticated methods [142]. This technological disparity represents one of the main structural barriers to the global transition toward more sustainable healthcare waste treatment systems.

EU-27 countries generated 101.4 million t of hazardous waste in 2018, representing 4.3% of total waste, with an 11.6% increase compared to 2010 [143]. This data reflects the growing challenge of hazardous waste management, including MW, across European healthcare systems.

Recyclable materials represent more than 40% of OR waste volume [144], yet standardized measurement protocols are lacking internationally, raising significant inconsistencies. Improper segregation rates remain high globally, with variations attributed to different training protocols and enforcement mechanisms [53,145].

The recent literature on MW management in surgical and hospital contexts reveals diverse strategies shaped by economic and regulatory settings. In high-income countries, targeted segregation, device reprocessing, and policy-driven audits yield measurable waste and cost reductions. LMICs report gains from pilot programs using training, audits, and digital tracking technologies. Lifecycle assessments and advanced performance metrics highlight that recycling quality, not just quantity, is essential for circular economy transitions. Evidence across contexts confirms that integrating regulatory frameworks, technology adoption, and behavior change achieves the most sustainable outcomes [84,146,147]. Table 4 presents key studies on MW management, organized thematically by country/region, method, and regulatory context, to facilitate cross-comparison of approaches and outcomes.

Priority research needs include comprehensive evaluation of emerging treatment technologies under different operational conditions. Long-term sustainability assessments of different treatment approaches require systematic investigation. Economic modeling of integrated waste management systems needs development to support evidence-based policy formulation.

## 6. Policies

As part of the Circular Economy Waste Package, the term “waste” is defined in Article 3 of the Waste Framework Directive (Directive 2008/98/EC, as amended by EU Directive 2018/851). The Court of Justice’s case law, which has opted to highlight the environmental protection features of the Directive, is crucial to the understanding of the concept of waste. The Court’s decision-making process is highly fact-specific and takes into account each individual case. By applying the definition of waste and finding that attempts to construe this meaning restrictively are incompatible with the Directive, the Court has elected to place an emphasis on the environmental protection provisions of the Directive [148]. Critically, while the legal framework is strong, there is limited evidence on Member States’ enforcement or how case law has influenced hospital-level waste practices.

**Figure 8:**
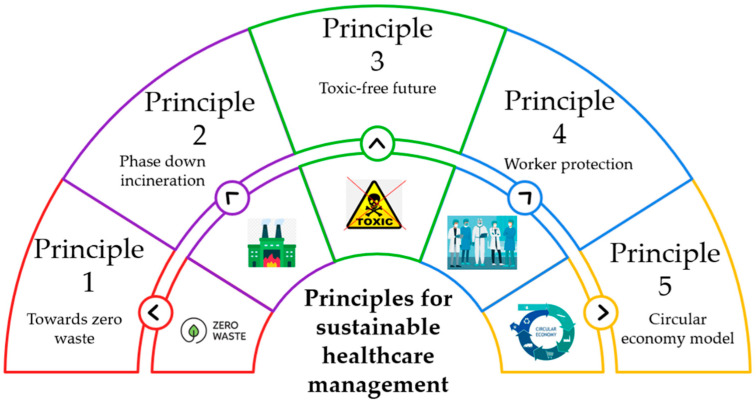
Strategic pillars for sustainable healthcare waste systems encompassing prevention, incineration phase-down, toxics elimination, worker protection and circularity.

Health Care Without Harm has recently established a comprehensive set of worldwide principles for sustainable management of MW (Figure 8). Yet, the implementation of these principles remains largely voluntary, with significant variability across countries and minimal tracking of adoption in clinical settings. Public health authorities should consider the widespread promotion and implementation of non-incineration technologies for the final disposal of MW as a means to mitigate environmental pollution and reduce the disease burden associated with incineration emissions [149].

EU published a directive in 2013 called “The European Eco-Management and Audit Scheme (EMAS)” with the goal of enhancing the environmental performance of businesses. Only companies that implemented EMAS successfully received the EU label. As of the initial months of 2012, 1007 German organizations were EMAS-registered or -certified. Most of these businesses have taken on the goal of improving the management of waste and utilization of resources as their primary objective. About 7% of these businesses are in the medical field [150]. By early 2025, EMAS included 4114 registered organizations and 15,815 sites [151], with approximately 653 active in waste management and disposal operations [152].

EU waste policy requires member states to align with European Commission (EC) directives. The EC recommends that MW be classified using Chapter 18 of the European Waste Catalogue (Decision 2000/532/EC), which standardizes descriptors for MW types [123]. But national transpositions differ in uniformity, and many lack harmonized data collection on waste categories or volumes generated in hospitals.

Despite a robust legal architecture (i.e., European Waste Catalogue, EWC/Decision 2000/532/EC [153]) and voluntary frameworks (Health Care Without Harm [154]), the reviewed evidence shows persistent intent–implementation gaps. First, mandated classification does not ensure correct point-of-care segregation: pre–post programs in Spain and India revealed substantial baseline misclassification, with improvement only after sustained training and feedback [69,70,123]. Circularity and decarbonisation goals coexist with continued reliance on incineration, although LCA from Iran indicates lower GHG burdens when recovery/chemical disinfection pathways displace thermal destruction [129]. Moreover, EU-compatible device reprocessing yields CO_2_ and cost savings in Scandinavia, yet uptake elsewhere is constrained by procurement/liability perceptions, an implementation rather than standards gap [58]. However, data transparency is uneven: NHS England mandates ≥95% reporting accuracy and segregation targets, whereas comparable national auditing remains limited in Romania and other EU settings [71]. While South Korea’s nationwide radio frequency identification enables end-to-end traceability [60], many European hospitals still rely on paper records despite European Waste Catalogue alignment [68,123]. Finally, heterogeneous outcome metrics (i.e., kg, cost, CO_2_-eq) hinder cross-jurisdiction comparison and policy learning [69,70,71,129].

The appropriate documentation in the collection, storage and disposal of MW in Romanian hospitals is based on:

− Order of the Ministry of Health, no. 1226/2012, for the approval of the Technical Norms regarding the management of waste resulting from medical activities and the Data Collection Methodology for the national database regarding waste resulting from medical activities [155];
− Framework Directive 2006/12/EC, which contains provisions for all types of waste, except those that are regulated separately by other directives [156];
− The Hazardous Waste Directive (Directive 91/689/EC) contains provisions on the management, recovery and correct disposal of hazardous waste [157];
− Own regulations and internal protocols developed by the unit.

While the legal basis is comprehensive, there is scant public data on compliance levels, nor on how municipal inspections or audits verify hospital practices. Recent Romanian legislative updates, harmonization with EU directives, and development of ward-specific protocols may enhance MW management by improving data collection, monitoring, regional analysis, transparency, stakeholder collaboration, and overall sustainability. Future directions include advanced segregation technologies, smart monitoring systems, life cycle assessment, continuous training, and integration of circular economy models. To date, pilot-scale implementation of smart bins or eco-procurement are systematically and comprehensively evaluated.

## 7. Conclusions

The present narrative review provides a comprehensive synthesis of current MW management practices, with particular attention to surgical settings and Romania’s specific context. Based on an integrated evaluation of the scientific literature, policy frameworks, and empirical insights, the key findings indicate that ORs are significant generators of MW, producing up to 30% of total hospital waste, while improper segregation rates remain high. Despite existing regulations, Romania lacks systematic audits, benchmarking tools, and feedback mechanisms for MW management in surgical wards. Although Romania aligns with EU legislation, implementation in surgical contexts remains largely descriptive and lacks performance metrics. International examples from the United Kingdom, the United States of America, South Korea, and the Netherlands highlight the benefits of structured segregation targets, digital tracking, staff training, and measurable outcomes.

Building on international best practices, several actionable policies can enhance surgical waste governance in Romania and similar healthcare systems. Strengthening enforcement of existing Romanian regulations through mandatory internal audits, national benchmarking, and public reporting frameworks is essential. Institutionalizing the role of dedicated waste managers in surgical facilities, prioritizing digital monitoring technologies and traceability systems for MW tracking and segregation efficiency, and expanding staff education programs across all professional roles with focus on compliance, classification accuracy, and behavioral change, represent critical interventions.

To support data-driven improvements and sustainable innovation, targeted research efforts should be directed toward longitudinal studies assessing the impact of education, digitalization, and policy interventions on waste volumes, costs, and emissions. The development of standardized metrics for MW evaluation across facilities and countries to allow for cross-national comparisons, the investigation of the feasibility and effectiveness of circular economy models in Romanian surgical contexts, including reuse and recycling technologies, and the exploration of stakeholder barriers and facilitators to adopting innovative waste management strategies in LMIC settings are essential research priorities.

## Figures and Tables

**Table 1:** Regulatory classification of medical waste targeting codes, hazard flags (*), and typical clinical items.

Waste Code	Official Description	MW Categories Resulting from Medical Activities
18 01 01	Sharps (except 18 01 03 *)	Disposable or unused sharps that have not come into contact with infectious material, such as needles, needle syringes, cannulas, catheters, pipettes, scalpel blades, and laboratory glassware. If contaminated with pathogens or biological fluids, these items are classified under 18 01 03 *. Sharps contaminated with toxic chemicals or hazardous substances fall under 18 01 06 *.
18 01 02	Human tissue and organs including blood and blood bags (except 18 01 03 *)	Waste generated from surgical and obstetrical procedures such as human tissues, organs, fetuses, placentas, blood, blood storage containers, and other biological fluids. When contaminated with infectious agents, these materials may be classified under 18 01 03 *.
18 01 03 *	Wastes whose collection and disposal is subject to special requirements in order to prevent infection	Infectious waste including materials contaminated with blood or other biological fluids; waste contaminated with bacteria, viruses, parasites, or microbial toxins; infusion sets with tubing; dialysis membranes; probes; gloves; disposable medical materials; dressings; compresses; laboratory materials; and other contaminated clinical supplies.
18 01 04	Wastes whose collection and disposal is not subject to special requirements in order to prevent infection	Non-infectious healthcare waste such as uncontaminated clothing, clean bed linens, properly plastered medical devices, thermally treated or decontaminated infectious waste, empty infusion bottles, and empty drug containers excluding cytotoxic or cytostatic pharmaceuticals.
18 01 06 *	Chemicals consisting of or containing hazardous substances	Chemical waste including solvents, acids, bases, halogenated solvents, organic and inorganic chemical substances, diagnostic laboratory waste, fixing and developing solutions, concentrated disinfectants, cleaning agents, and formaldehyde-containing solutions.
18 01 07	Chemicals other than those mentioned in 18 01 06	Non-hazardous chemical products such as diluted disinfectants (e.g., weak sodium hypochlorite solutions), cleaning agents without hazardous labeling, and waste from medical equipment containing small amounts of non-hazardous chemicals.
18 01 08 *	Cytotoxic and cytostatic medicines	Waste generated from cytotoxic and cytostatic pharmaceuticals used in chemotherapy and oncology treatments, including expired or unused antineoplastic drugs and materials contaminated with such substances.
18 01 09	Medicines other than those mentioned in 18 01 08	Non-cytotoxic pharmaceutical waste such as expired or unused medicines that do not contain cytotoxic or cytostatic substances.
18 01 10 *	Amalgam waste from dental care	Dental amalgam waste containing mercury, including amalgam fragments, used amalgam capsules, photopolymerizable composite materials, dental crowns and bridges, and extracted teeth containing amalgam fillings.

MW, medical waste.

**Table 2:** Color-coded segregation matrix for healthcare waste: EWC codes, container types, and bilingual data. The obligation to display waste collection instructions is marked with the * symbol.

Displayed in Romanian	Translated Data	Official Description	Color Symbol
18.01.01/18.01.03 *—(recipient plastic galben) = Deșeuri înțepătoare-tăietoare, ascuțite	18.01.01/18.01.03 *—(yellow plastic container)—Sharp-pointed, cutting waste	18 01 01—Sharps (except 18 01 03 *)/18 01 03 *—Wastes whose collection and disposal is subject to special requirements in order to prevent infection	yellow
18.01.03 *—cutia Actis—box = Deșeuri infecțioase	18.01.03 *—Actis box—box—Infectious waste	18 01 03 *—Wastes whose collection and disposal is subject to special requirements in order to prevent infection	yellow
18.01.04—sac negru sau galben = flacoane de perfuzie, scutece de la pacienți neinfecţioşi, flacoane din sticlă de antibiotice şi perfuzii	18.01.04—black or yellow bag—Infusion bottles, diapers from non-infected patients, glass bottles of antibiotics and infusions	18 01 04—Wastes whose collection and disposal is not subject to special requirements in order to prevent infection	yellow or black
15.01.10 *—ambalaje cu inscripția toxic, inflamabil, coroziv	15.01.10 *—Packaging with the label toxic, flammable, corrosive	15 01 10 *—Packaging containing residues of or contaminated by hazardous substances	-
15.01.01—hârtie, carton	15.01.01—Paper, cardboard	15 01 01—Paper and cardboard packaging	blue
15.01.07—sticle, borcane	15.01.07—Glass, jars	15 01 07—Glass packaging	green
15.01.02—material plastic, flacoane apă	15.01.02—Plastic material, water bottles	15 01 07—Glass packaging	yellow
20.03.01—deșeuri menajere	20.03.01—Household waste	20 03 01—Mixed municipal waste	green

**Table 3:** Summary of key studies on medical waste generation, management interventions, and lessons learned.

Country/ Region	Study Context	Intervention/Data	Key Findings	Lessons Learned	Ref.
China, Greece, Lebanon, South Korea, England, Türkiye	MW generation trends	China: 0.7–1.5 kg/capita/year; Greece: 0.70 kg; Lebanon: 1.42 kg; South Korea: 7.2 kg; England: ~156,000 t/year; Türkiye: ~143,500 t/year (forecast >211,000 t by 2030)	MW generation varies widely;	Urbanization and healthcare expansion are key drivers	[97]
Spain	Surgical department	Improved segregation protocols and staff training	Carbon footprint reduced by ~85% weekly	Behavioral interventions and staff training significantly improve segregation performance	[105]
Global	Smart MW management	IoT-enabled monitoring systems	Real-time tracking improves collection efficiency and regulatory compliance	Digital monitoring enhances traceability and operational efficiency	[59]
Global	Automated segregation	AI-based SmartMedWaste system	Waste classification accuracy reached 97.93%	Automation can reduce human error and improve sorting efficiency	[106]
Ethiopia/Sudan/Brazil	Waste handlers exposure	Occupational risk studies	High prevalence of needlestick injuries among MW handlers	Protective equipment and staff training are critical for occupational safety	[107]
China	Pandemic waste logistics	Optimization model for MW transport	Cross-regional transportation reduced infection risk and operational costs	Adaptive logistics systems are crucial during epidemics	[104]
Iran (Tehran hospitals)	Hospital waste management	Segregation and on-site disinfection	Costs reduced up to 50% and pollutant emissions lowered	Segregation combined with local treatment improves environmental and economic performance	[110]
Iran (Isfahan hospitals)	Life-cycle assessment	Alternative treatment scenarios	Composting and recovery reduced emissions compared with incineration	Circular-economy approaches improve environmental sustainability	[129]
Romania	Pharmaceutical waste disposal	Public survey and pharmacy management analysis	Low compliance with proper disposal of pharmaceuticals	Public education and clearer regulatory frameworks are required	[56,136]

MW, medical waste; IoT, internet of things; AI, artificial intelligence; Ref, references.

**Table 4:** Comparative overview of key studies on medical waste management by country, intervention, and policy context.

Country	Setting	Method/Intervention	Key Outcome	Policy Context	Thematic Focus	Ref.
USA (Minneapolis VA)	OR waste audit	Composition analysis	Audit revealed 84.5% of operating room waste was general, indicating high potential for diversion from regulated streams	Hospital practice norms	Baseline metrics	[32]
USA (Magee-Womens)	OR & L&D	RMW reduction program	Reduction in regulated medical waste by 47%, with $89,000 annual cost savings through improved segregation	Institutional policy	Training; process redesign	[55]
Netherlands	OR	Quantify unused disposables	Identified €676.49 per case in unused sterile disposables, highlighting procurement inefficiencies	Procurement policy	Waste prevention	[87]
Spain	Surgical department	Training + protocols	Implementation of training and standardized segregation protocols reduced medical waste by 6.2% per month and saved €125,000 annually	Hospital policy	Segregation; emissions	[69]
India	Teaching hospital	Training + monitoring	Training and continuous monitoring reduced segregation deficiencies from 1.10% to 0.03%	National BMW rules	Training; QA	[70]
UK	NHS England	HTM 07-01 audits	Policy-driven audits achieved ≥95% reporting accuracy across facilities, improving governance and oversight	National targets	Governance	[71]
Republic of Korea	National MW	RFID tracking	Nationwide adoption of RFID-enabled tracking ensured full traceability of medical waste from generation to disposal	National regs	Digital traceability	[60]
Iran	Hospitals	LCA scenarios	Life cycle assessment showed greenhouse gas savings when recovery and recycling scenarios were applied over incineration	Municipal policy	CE strategies	[129]

OR, operating room; L&D, labor and delivery; RMW, regulated medical waste; BMW, biomedical waste; QA, quality assurance; NHS, National Health Service; HTM, Health Technical Memorandum; MW, medical waste; RFID, radio frequency identification; LCA, life cycle assessment; CE, circular economy.

## Data Availability

The authors express their gratitude to the University of Oradea, Oradea, Romania, for supporting the APC.

## Abbreviations

The following abbreviations are used in this manuscript:

MW	Medical Waste
EU	European Union
EWC	European Waste Catalogue
RMW	Regulated Medical Waste
STs	Surgical Technicians
SDG	Sustainable Development Goals
OR	Operating Room
GHG	Greenhouse Gases
DSA	Digital Subtraction Angiographies
LMIC	Low- and Middle-Income Countries
EMAS	European Eco-Management and Audit Scheme
AI	Artificial Intelligence
IoT	Internet of Things
LCA	Life cycle assessment
